# Assessing alternative lake management actions for climate change adaptation

**DOI:** 10.1007/s13280-024-02039-y

**Published:** 2024-06-14

**Authors:** Shajar Regev, Yohay Carmel, Gideon Gal

**Affiliations:** 1 Kinneret Limnological Laboratory, Israel Oceanographic and Limnological Research, 14950000 Migdal, Israel; 2 Faculty of Civil and Environmental Engineering, The Technion-Israel Institute of Technology, 3200003 Haifa, Israel

**Keywords:** Climate change, Cyanobacteria, Lake ecosystem, Lake Kinneret, Lake management, Uncertainty

## Abstract

Lake management actions are required to protect lake ecosystems that are being threatened by climate change. Freshwater lakes in semiarid regions are of upmost importance to their region. Simulations of the subtropical Lake Kinneret project that rising temperatures will cause change to phytoplankton species composition, including increased cyanobacteria blooms, endangering lake ecosystem services. Using lake ecosystem models, we examined several management actions under climate change, including two alternatives of desalinated water introduction into the lake, hypolimnetic water withdrawal, watershed management changes and low versus high lake water level. To account for prediction uncertainty, we utilized an ensemble of two 1D hydrodynamic—biogeochemical lake models along with 500 realizations of meteorological conditions. Results suggest that supplying desalinated water for local use, thus releasing more natural waters through the Jordan River, increasing nutrient flow, may reduce cyanobacteria blooms, mitigating climate change effects. However, these results are accompanied by considerable uncertainty.

**Supplementary Information:**

The online version contains supplementary material available at 10.1007/s13280-024-02039-y.

## Introduction

Climate change is projected to bring about increased air temperatures globally and reduced precipitation in subtropical regions. According to the CORDEX-CORE regional climate projections, in the eastern Mediterranean region, under a business-as-usual Representative Concentration Pathway (RCP8.5), air temperature is expected to gradually increase by 2.5 °C in 50 years compared to the average temperature during 2010–2020. In addition to temperature rise, significant reduction in precipitation, and evaporation increase is projected for the Eastern Mediterranean (Zittis et al. 2022).

Semiarid ecosystems are threatened both by climate change and increasing water demand for agriculture and for growing populations (Çolak et al. 2022). This renders maintaining water quality of freshwater lakes in these regions of upmost importance. The expected temperature increase and precipitation decrease may threaten lake water quality through lake elevated temperatures and nutrient availability changes. Elevated temperatures are anticipated to induce cyanobacteria blooms (Jankowiak et al. 2019). Changes in nutrient availability have unclear effect; nonetheless, cyanobacteria blooms are increasing in occurrence and intensity in lakes worldwide due to their wide temperature tolerance and ability to efficiently use nitrogen and phosphorus (Sinha et al. 2012), resulting in water quality issues due to production of cyanotoxins (Plaas and Paerl 2021).

Located in the semiarid eastern Mediterranean, Lake Kinneret is undergoing changes such as increased cyanobacteria blooms over the last 30 years. These changes are attributed to climate change and nutrient reduction (Hadas et al. 2015). Model simulation of Lake Kinneret projects that these changes will continue to affect the phytoplankton species composition, endangering lake ecosystem services (Regev et al. 2024). The projected air temperature rise will decrease oxygen availability, leading to a decrease in ammonium concentrations (Regev et al. 2024). This, together with reduced nutrient inflow, will cause an increase in N fixing cyanobacteria blooms and decrease blooms of the dinoflagellate and green phytoplankton group, reducing overall primary production. This means that unlike many lake ecosystems which are expected to undergo eutrophication, Lake Kinneret, and possibly other subtropical lakes, will have reduced nutrient availability. In order to maintain a stable ecosystem, it is plausible that nutrients may need to be added rather than reduced.

Lake management actions are required to restore and conserve lake ecosystems that are being threatened by climate change and anthropogenic pressures. Actions should be taken to maintain a stable lake ecosystem, counteracting the effects of rising water temperature. There is a variety of viable tools available to lake managers to tackle both the effect of rising temperature and reduced precipitation, as projected under climate change in semiarid regions. Most of these lake management tools aim to control nutrient availability; they include control of nutrients sources in the watershed, lake nutrient control through hypolimnetic withdrawal, phosphorus precipitation and hypolimnion aeration (Yasarer and Sturm 2016; Nürnberg 2020). Additional management actions, more hydrological in nature, are modification of residence time (Olsson et al. 2022) and lake level control. The impact of using these tools, where they are applicable, is not clear and requires testing.

Modeling is the ideal tool to explore the impact of management actions. The application of coupled hydrodynamic–biogeochemical lake models is an established approach for studying lake ecosystem responses to external drivers such as climate change and management actions (Soares and do Carmo Calijuri 2021). The use of ecosystem models provides a range of benefits, but such models suffer from limitations and weaknesses.

One of the main weaknesses of ecosystem models is the results uncertainty, stemming from multiple sources. A main source of uncertainty arises from the models themselves, mainly due to the challenge of simulating highly complex systems. These intricate processes cannot be fully described by model equations, so simplifications are made, resulting in model inaccuracy and uncertainty (Puy et al. 2022). Applying multiple, independently developed models, i.e., an ensemble modeling approach, can deliver information about the inherent model uncertainty. It can strengthen the confidence in the projections when the models agree on trends and point to areas of higher uncertainty when projections diverge, reflecting models’ structural uncertainty. Conveying the mean and range of the projections can reduce uncertainties in individual model projections. Ensemble modeling has been recently introduced to lake modeling studies and is fast becoming ‘best practice’ among marine ecosystem modeling groups, particularly when addressing large-scale questions such as the effects of climate change (Lotze et al. 2019; Geary et al. 2020).

Another source of uncertainty in climate change studies is associated with the projected climate change, also termed as deep uncertainty (Walker et al. 2013). It is uncertain which climate change scenario is going to materialize. In addition, lake models require time series of meteorological conditions, but the translation of a climatic scenario into such a time series is highly uncertain. There are infinite possibilities of weather conditions that can occur under a single climate scenario; however, general or regional climate model output produces only a single time series. A weather generator can be used to bridge the gap between the typical output of climatic models and the required input to lake models. A weather generator (WG) is a computer software that produces synthetic time series that resemble realistic meteorological conditions. The WG creates numerous meteorological time series that follow climate change scenarios while maintaining variability and patterns of existing weather conditions (Ailliot et al. 2015). These meteorological time series are called hereafter realizations of a climate scenario. The lake model is then run using each of these realizations as forcing data, producing many projections that enable evaluating the uncertainty resulting from weather variability. A WG coupled with lake modeling has been used recently (Schlabing et al. 2014; Regev et al. 2024), producing predictions that better convey the trends and uncertainty resulting from climate change scenarios.

In this study, we follow the modeling methods of Regev et al. (2024) and explore potential management actions for mitigating the impact of climate change on the subtropical Lake Kinneret. We do so by applying a two-member ensemble of 1D lake ecosystem models to the lake. We explore the efficiency of various management actions under RCP8.5-based meteorological conditions of gradual air temperature rise over a period of 49 years, derived by WG.

## Materials and methods

The methods used in this study were mostly identical to those described in Regev et al. (2024). We therefore describe the methods briefly but provide detailed information on the methodology that differs from the earlier work.

### Study site

Located in northern Israel, Lake Kinneret (Sea of Galilee, 210 m below sea level) is a meso-eutrophic lake, covering an area of 168 km^2^ and has a maximum depth of 44 m. The lake is the only natural freshwater lake in Israel and provides critical ecosystem services including drinking water, fisheries and recreation, as well as cultural and religious services. Lake stratification lasts about 10 months a year (March–January) with an anoxic hypolimnion. The Jordan River is the main stream that flows into the lake, contributing about 75% of the inflow. Analysis of the nutrient loads from the river into the lake and their mass balances indicate that the Jordan River introduces most of the N input but only one-third of the P input (Be’eri-Shlevin et al. 2023).

### The models

We applied a two-member ensemble of 1D models of Lake Kinneret for this study. The models included: (1) GOTM-WET (Schnedler-Meyer et al. 2022) called hereafter WET, and (2) DYRESM-CAEDYM (DYCD) (Hipsey and Hamilton 2008). Both models are process based, comprised of a hydrodynamical model coupled to a biogeochemical model; both calculate the lake’s hydrodynamic properties, nutrient concentration, plankton biomass and more. The biotic structure in both WET and DYCD included five phytoplankton and three zooplankton groups. The phytoplankton groups were configured according to their different nature and seasonal growth: (1) Dinoflagellates (Dino) (2) Nitrogen fixing cyanobacteria (NfixCyano) (3) *Microcystis spp.* (CyanoMC) (4) Diatoms and (5) Green algae including phytoplankton that do not belong to groups 1–4. Zooplankton were configured based on three functional groups: (1) Herbivorous zooplankton (HerbZoo) (2) Microzooplankton (MicroZoo) and (3) Predatory zooplankton (PredZoo). WET is based on closed nitrogen and phosphorus cycles across the biotic and abiotic lake components, whereas DYCD also tracks the carbon cycle. Both models were calibrated and validated for Lake Kinneret (Gal et al. 2009; Regev et al. 2023). For further details, see Regev et al. (2024).

### Model inputs

#### Meteorological conditions

For meteorological condition inputs, we used the same time series that was produced by the weather generator ‘WeatherCop’ (Schlabing 2021) in Regev et al. (2024), which follows a gradual air temperature increase of 2.5 °C over 50 years. This scenario relates to RCP8.5 according to the CORDEX-CORE regional climate projections (Zittis et al. 2022). A total of 500 realizations that follow the climate change scenario while maintaining variability and patterns of local weather conditions were used for the simulation of each management action. The realizations span over 50 years, starting from October 1st, 2020, which is the onset of the hydrological year, essential for producing Jordan River flows. Lake models were run starting February 1st, 2021, when the water column is mixed, so the total length of the simulations was 49 years.

#### Hydrological model, stream flows, nutrient loads and water withdrawals

The forcing data for the models included daily inflows and nutrients from streams. Precipitation generated by WeatherCop was translated into flows in the Jordan River based on the hydrological model HYMKE (Rimmer and Salingar 2006). Nutrient concentrations in the Jordan River were calculated as a linear approximation of (1) mean daily, day of the year specific, nutrient concentration (from historical data) and/or (2) of the flow volume (see Appendix S1). Stream flows and nutrient loads vary between management actions—see Sect. 'Management actions.' Lake level in all scenarios was managed by water withdrawals from the lake to sustain a stable level while maintaining natural seasonal fluctuations of ± 2 m (see Appendix S2).

### Management actions

Lake management actions are derived from the state of the watershed and the water demands in the region. Lake Kinneret watershed includes the agricultural Hula Valley. Changes to the watershed during the last three decades have most likely impacted Lake Kinneret. Among these changes are the establishment of the shallow Lake Agmon (Hambright and Zohary 1998) and the ongoing exploitation of many water springs for irrigation, thought to have reduced the Jordan River flow (Wine et al. 2019). Mitigating management actions can potentially include altering the flow through Lake Agmon and reducing spring water exploitation. Reduction of anthropogenic nutrient loads has already largely been achieved and hence was not tested.

Following five consecutive drought years between 2013 and 2018 in northern Israel, the Kinneret lake level dropped considerably by 3.5 m. Lake Kinneret is a critical source of drinking water to Israel and to the Kingdom of Jordan; for this reason and additional policy considerations, the Israeli Water Authority launched a project to introduce desalinated water from the Mediterranean Sea into the lake, despite the high energy consumption involved. See Appendix S3 for information on the desalination project. The inflow of desalinated water into the lake presents a unique management action for mitigating climate change effect on Lake Kinneret.

A total of twelve scenarios were defined as combinations of management actions (Table 1). In all management scenarios, including a no-action scenario, we used the 2.5 °C over 50 years gradual temperature increase meteorological input. The scenarios include changes in quantity and quality of the water flowing into the lake. Following is a description of the scenarios: (1) Increased water flow through Lake Agmon, which is a source for pit soil nutrients, thus doubling the amount of nutrients outflowing from Lake Agmon into the Jordan River (Table 2); the added nutrients were evenly distributed through the year. (2) An addition of 150∙10^6^ m^3^ desalinated water per year carried up north to the Hula valley, supplying the local water consumption and thus allowing release of natural spring waters into the Jordan River. The addition was from February through to June every year when a surplus of desalination capacity is expected. Higher flows carry more nutrients, and in addition, nutrient concentration is higher as it is correlated with a higher Jordan flow (see Sect. 0). (3) Desalinated water is input to the lake via the ephemeral Tsalmon Stream, with the same volume and timing as the previous scenario. In this scenario, the water flows a shorter route before reaching the lake and thus the assumption was made that these waters have zero nutrients and therefore the nutrient inputs are identical to the no-action scenario. The Tsalmon water temperature was set to rise from 20 °C in February to 25 °C in June. (4) Hypolimnetic withdrawal scenarios, which reduce nutrient buildup during the stratified period, were conducted from a depth of 28 m which is below the thermocline most of the year. Hypolimnetic withdrawal was combined with the two desalinated water scenarios. (5) All these scenarios were run once while the lake was maintained at high lake level (L1) and once when the lake level was 3 m lower (L2).

**Table 1:** Management scenarios

Name	Flow and nutrients scenarios	Initial lake level [masl]	
		− 211 (L1)	− 214 (L2)
No-action	No action	No-action-L1	No-action-L2
AN	Double Agmon Nutrients outflow (by reduced Lake Agmon residence time)	AN-L1	AN-L2
DJ	Desalinated water added through the Jordan River (Nutrients amplified by flow), 150Mm^3^	DJ-L1	DJ-L2
DT	Desalinated water through Tsalmon (0 nutrients), 150Mm^3^	DT-L1	DT-L2
DJHW	Desalinated through Jordan + Hypolimnetic Withdrawal	DJHW-L1	DJHW-L2
DTHW	Desalinated through Tsalmon + Hypolimnetic Withdrawal	DTHW-L1	DTHW-L2

**Table 2:** Added nutrient outflow from Lake Agmon into Jordan River. Data from Barnea et al. (2012) and Hula project unpublished data

Nutrient	Added nutrient outflow from Lake Agmon
	Tonne per year
N Particulate Organic Matter (NPOM)	10
${\text{NH}}_{4}^{ + }$	22
P Particulate Organic Matter (PPOM)	2.2
${\text{PO}}_{4}^{3 - }$	0.5

### Model outputs and analysis

The responses of the two lake models to the twelve alternative management scenarios were evaluated using model outputs, including the stratification characteristics, residence time, and several chemical and biological state variables. Stratification characteristics included stratification days and thermocline depth and residence time was calculated using the age structure method set forth by Gilboa et al. (2022). The analysis of water temperature, O_2_, ${\text{PO}}_{4}^{3 - }$, ${\text{NH}}_{4}^{ + }$ and ${\text{NO}}_{3}^{ - }$, was reduced to the mean epilimnion (above 11 m) and hypolimnion (below 28m) values. The hypolimnion nutrients were calculated only during the stratified period as determined by stratification days. Phytoplankton biomass and zooplankton biomass were analyzed as total epilimnion biomass. To examine the effect of the various scenarios, the percentage of change relative to the relevant reference simulation was calculated for the last year of the simulation, for each variable according to Eq. 1. This was performed with three different references: (1) All scenarios were compared to the no-action scenario, this was done only at high lake level (L1). (2) All scenarios at a low lake level (L2) were compared to high lake level (L1). (3) Hypolimnetic withdrawal scenarios (DJHW, DTHW) were compared to DJ & DT. The percentage of change (Eq. 1) was applied to all 500 realizations to obtain variability associated with meteorological variability. The variability is presented as split violin plots, where each model is represented by a half violin. In these plots, if both distribution peaks are above (or below) zero it means both models predict the same trend.

1 $$\% Change = 100 \cdot \left( {\frac{{\overline{{var_{Scen} }} - \overline{{var_{Ref} }} }}{{\overline{{var_{Ref} }} }}} \right)$$

Top bar indicates mean over the 49th year.

As an additional, holistic evaluation of management scenarios, we calculated the expected value of the Kinneret Sustainability Index (KSI) for each scenario. Gal and Zohary (2017) introduced the KSI in order to evaluate ecosystem changes in Lake Kinneret in relation to a reference period, i.e., the KSI is a measure of the ecosystem similarity to a reference state. The KSI is based on comparing the nine following ecosystem variables to a reference state: (1) Secchi depth, (2) total suspended solids, (3) total nitrogen (TN), (4) total phosphorus (TP), (5) chlorophyl *a,* (6) primary production, (7) cyanobacteria biomass, (8) *Peridinium gatunense* biomass and (9) predatory zooplankton biomass. Median values that reflect conditions during the period between 1990 and 2020, as generated by the models, were used to set the reference values. These reference values are also termed ‘Unchanging climate’. Each variable is ranked with a value between 10 and 100 representing similarity to the reference state. The weighted average of the nine components yields a single KSI value that represents how close the ecosystem is to the reference state. Higher KSI values represent a better ecosystem state; ‘better’ in the sense that it is closer to the ecosystem conditions in the last 30 years. KSI values > 60 are considered acceptable, values 60 ≥ KSI ≥ 50 are unacceptable and KSI < 50 is considered highly unacceptable. To regard for variability, we calculated the probability of KSI receiving an unacceptable rank (< 50). For more details, see Appendix S4.

## Results

**Fig. 1:**
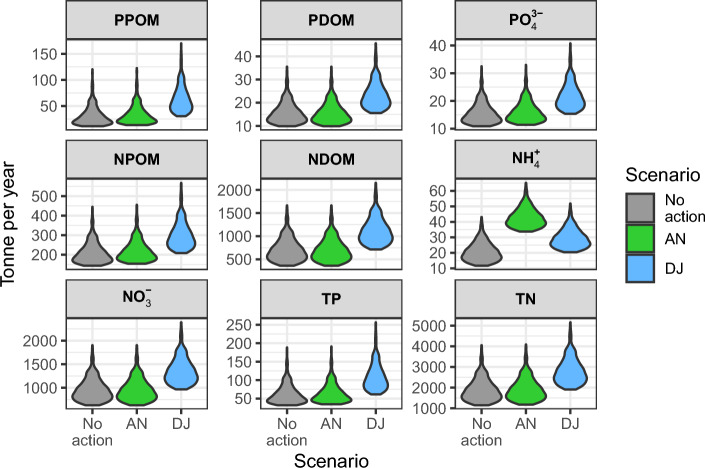
Nutrients inflows in three scenarios. No-action scenario (gray) also includes DT & DTHW scenarios having the same nutrient inflow. DJ (blue) includes also DJHW. AN scenario is in green. Variability is caused by precipitation variability produced by WG. Nutrient components abbreviations: PPOM & NPOM, P & N particulate organic matter; PDOM & NDOM, P & N dissolved organic matter; TP & TN, total P and total N

The two models were run with twelve combinations of management scenarios, each with 500 realizations of meteorological conditions. The difference between scenarios was most pronounced when flows were directed through the Jordan River (Fig. 1) in which the nutrient concentration was significantly higher. Relative to the no-action scenario, DJ inflows contained 1.8 and 1.5 times TP and TN loads, respectively. These values and all values hereafter refer to the median of the 500 repetitions.

### Lake level effect

**Fig. 2:**
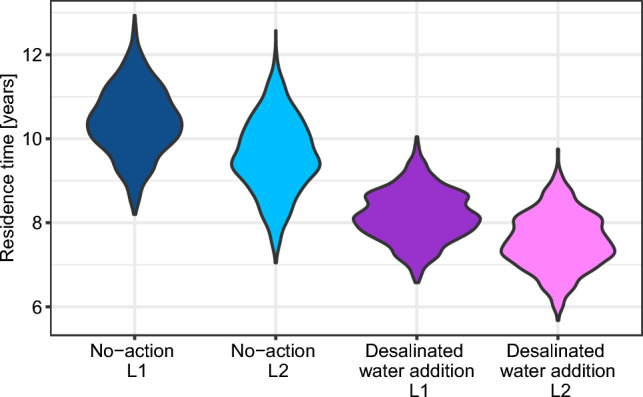
Residence time in the last year of the simulation for scenarios that have different inflow and lake volume. No water addition in blue (no-action, AN). Water addition in purple (DJ, DT, DJHW, DTHW). Low lake level (L2) in lighter hue

Maintaining low lake level (L2) reduced residence time by about 0.75 year in the final year of the simulation compared to L1 (Fig. 2). Lake level had an effect on a number of variables, but the effects were consistent across scenarios. Both models agreed that epilimnion temperature will be colder in L2 relative to L1, but showed different trends for hypolimnion temperature, epilimnion oxygen and other variables. Overall, 13 variables out of 20 had different trends between models. We delve into the lake level effect in Appendix S4. The rest of the results analysis hereafter includes both lake levels together.

### Management scenarios

#### Hypolimnetic withdrawal (HW)

**Fig. 3:**
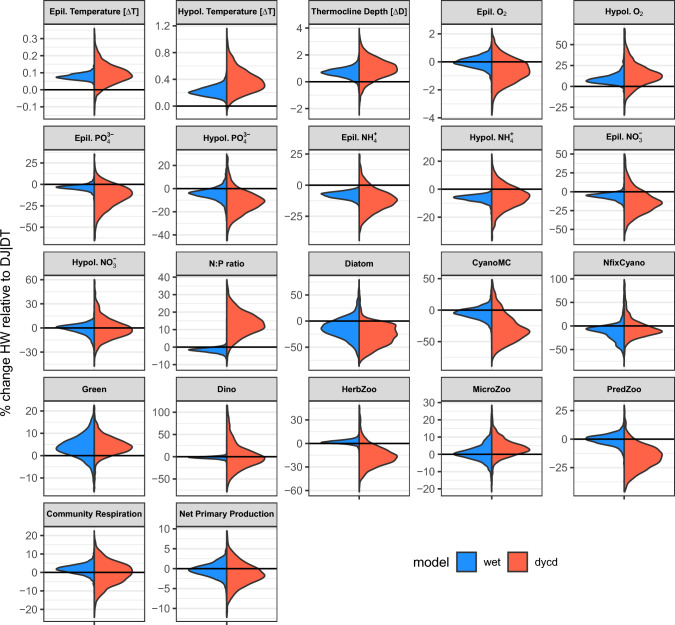
Hypolimnetic withdrawal effect. Change in percent during the last year of the run for the DJHW and DTHW scenarios relative to the corresponding scenarios without hypolimnetic withdrawal (DJ & DT). Temperature and thermocline depth show absolute difference, not %. For visibility, density is scaled to have the same width

The hypolimnetic withdrawal scenarios (DJHW, DTHW) resulted in increased water temperature as colder water was removed (Fig. 3). In addition, the thermocline depth increased by 0.9 m (ensemble mean), as displayed by both models, but the turnover day did not change (result not shown). An 8% reduction in hypolimnetic ${\text{PO}}_{4}^{3 - }$ was achieved and during turnover the epilimnion received less ${\text{PO}}_{4}^{3 - }$ and thus reduced the winter blooms of diatoms by 20%. According to the DYCD model, CyanoMC, herbivore and predatory zooplankton biomass was also lower. In 19 of the 22 variables examined, we found the same trends, in both models, resulting from hypolimnetic withdrawal.

#### Introducing desalinated water into the lake

**Fig. 4:**
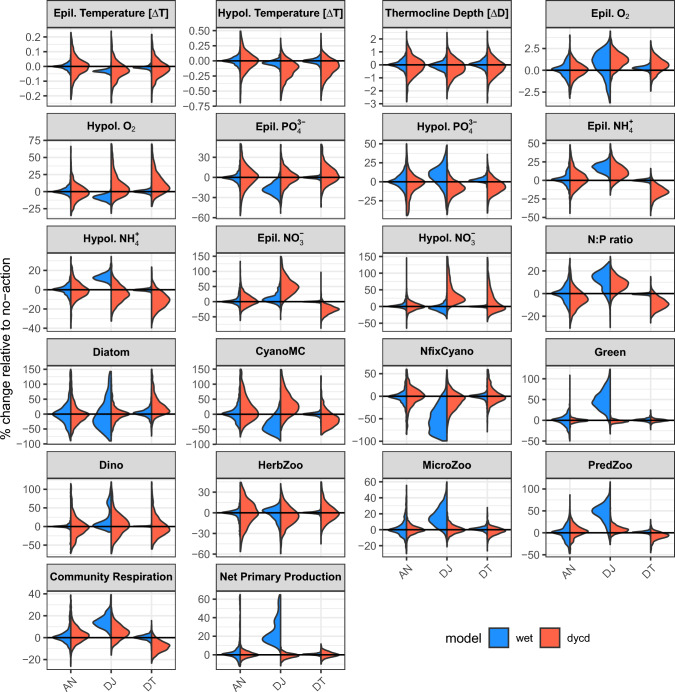
% change relative to the no-action scenario of the AN, DJ, & DT scenarios during the last year of the simulations. Temperature and thermocline depth show absolute difference, all other graphs show relative difference in %. For visibility, density is scaled to have the same width

Introducing more water into the lake reduced residence time by approximately two years (Fig. 2) but apart from that, the two inflow route options (DT,DJ) differ considerably in their effect. Hypolimnetic withdrawal (HW) scenarios had minimal interaction with the desalinated water introduction scenarios, so DTHW & DJHW are not presented as their responses are similar to the DT & DJ. The DYCD model suggested that introducing desalinated water through the ephemeral Tsalmon Stream (DT) will increase hypolimnion oxygen by 18%, decrease epilimnion ${\text{NH}}_{4}^{ + }$ & ${\text{NO}}_{3}^{ - }$ by 20% & 31%, respectively, and will eventually cause a 34% reduction in CyanoMC (Fig. 4). In contrast there was only a negligible effect projected by the WET model in the DT scenario.

WET model runs predicted that the highest effect would be obtained when desalinated water is introduced to the Hula valley, releasing natural, nutrient-rich waters to the Jordan River (DJ) (Fig. 4). The effect included almost all biogeochemical variables resulting in changes in the hypolimnion and epilimnion. In the hypolimnion, there was a 6% reduction in O_2_ and increase in ${\text{PO}}_{4}^{3 - }$ & ${\text{NH}}_{4}^{ + }$ (8% and 10%, respectively). In the epilimnion, O_2_ increased by 1% (-1.3%, 2.4% are the 10th and 90th percentiles), ${\text{NH}}_{4}^{ + }$ increased by 13% and ${\text{PO}}_{4}^{3 - }$ decreased by 18%. The biotic response included increased green phytoplankton biomass by 51%, and reduction of both N fixing cyanobacteria (62%) and CyanoMC (31%) biomass. Total primary production was higher (23%) and so was the microzooplankton (16%) and predatory zooplankton (48%) biomass. In contrast, the DYCD model projected only minor changes in the biotic response, except for CyanoMC which showed a 10% increase in biomass.

#### Double Agmon Nutrients outflow (AN)

Diverting more water through the shallow Lake Agmon, doubling the nutrients coming out of it, had a negligible effect. The percent change of all variables relative to the no-action scenario was around zero (Fig. 4).

### Kinneret Sustainability Index (KSI)

**Fig. 5:**
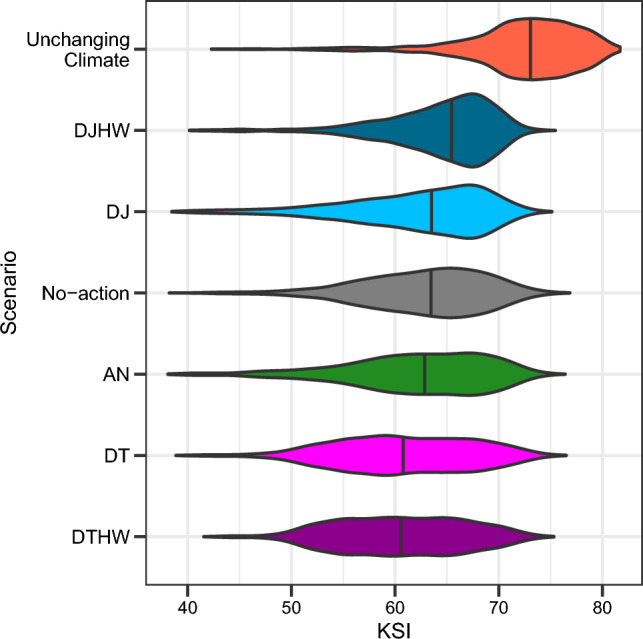
KSI distribution for each management action, including baseline which represents lake conditions without climate change. All models and lake levels are included. Black Line represents the median value. Management actions are ordered by KSI median values. All violins have the same area

The distribution of KSI values over the 500 realizations for the two models, across various scenarios and two lake levels, showed that the variability due to meteorological conditions and between the two models is much higher than the effect of any specific management action (Fig. 5). Though most KSI values projected were above 60 which is considered acceptable, all management actions had much lower KSI values than an unchanged climate scenario. When assessing the action scenarios including the two models and two lake levels together, the DJ & DJHW scenarios were the only scenarios in which more than a half of the instances was better than not taking any action (Fig. 5, Table 3). The DJHW scenario had the lowest probability (P <  = 0.04) of KSI < 50. Components affecting the KSI score, lake level effect and difference between models are provided in Figs. S1 and S2 in Appendix S5.

**Table 3:** Measures based on KSI values in the last year of the model runs for the two lake levels together. For the two models together: Median KSI and Probability for KSI < 50 (highly unacceptable value). By model: Is KSI better than no-action (↑ = better, ↓ = worse, →  = same) calculated as median of the difference from the no-action scenario. ‘Both’ column is for both models and both lake levels

	Median KSI	Probability of KSI < 50	KSI in relation to no-action		
No-action	63.5	0.03	**WET**	**DYCD**	**Both**
AN	62.9	0.07	↑	↓	↓
DT	60.7	0.05	→	↓	↓
DTHW	60.6	0.03	↓	↓	↓
DJ	63.6	0.05	↑	↓	→
DJHW	65.5	0.03	↑	↑	↑

## Discussion

We simulated a number of potential management actions for mitigating climate change effects on a subtropical lake using ensemble of two lake models and 500 realizations of meteorological conditions. The importance of subtropical lakes to their region and the use of ensemble modeling and a weather generator to evaluate potential actions for mitigating the impacts of climate change, while revealing projection uncertainty, makes this work significant. The results suggest that the DJ & DJHW management actions have the potential to counteract some climate change effects such as reduced ammonium, N:P reduction and NfixCyano increase.

### Mitigating climate change impact on subtropical lake ecosystem

Climate change is expected to decrease oxygen and ammonium levels in the epilimnion during summer and thus enhance N fixing Cyanobacteria blooms. Concurrently, overall primary production is expected to decline, mainly due to a reduction in the Dino phytoplankton group but also in the green phytoplankton (Regev et al. 2024). Can management actions increase oxygen and ammonium? The two desalination water addition scenarios add the same water volume with or without nutrients. When comparing these two scenarios, it is apparent that nutrient-rich water produces a substantial response while desalinated water generates only a minor response. The response to the nutrient-rich water scenario includes an increase in oxygen and ammonium concentration, hence counteracting climate change effects. The biotic response included decrease in NfixCyano and increase in Green phytoplankton, also counteracting climate change effect. A strong mitigation effect of the biotic response was projected by the WET model, and the ensemble mean also shows this effect. Median KSI values are highest for these scenarios, meaning that these scenarios have a higher probability to maintain the ecosystem near its current state. This finding is in accordance with the preliminary notion that in subtropical lakes nutrients should be added, rather than reduced, in order to mitigate climate change impact on the lake ecosystem. In contrast, introducing desalinated water through the Tsalmon stream results in decreased ammonium through increased nitrification and dilution (according to the DYCD model), thus intensifying climate change effects.

The models suggested that diverting more water through Lake Agmon, doubling its nutrient contribution, has a minor effect on the ecosystem of Lake Kinneret, since the nutrient contribution of Lake Agmon is relatively low. In contrast to nutrient addition, the aim of hypolimnetic withdrawal is a reduction of nutrient concentration buildup in the hypolimnion which is the main source of nutrients to the epilimnion following lake turnover and stratification. Indeed, reduced nutrients were achieved which reduced CyanoMC and NfixCyano blooms, while the Green phytoplankton biomass increased. This biotic response moderately counteracts climate change effect. However, in addition to removing hypolimnetic nutrients, hypolimnetic withdrawal also removed colder water and thus contributed to the overall warming of the lake, possibly amplifying climate change effect. Another drawback of hypolimnetic withdrawal is that the anoxic hypolimnion contains sulfides (Hadas and Pinkas 2014), so water must be treated before used.

Droughts are increasing in frequency and intensity in semiarid regions that reside by an ocean, for example, Spain, Australia and California (Berbel and Esteban 2019; Morote et al. 2019), and as a result there is an increase in the production of desalinated water. Our study contributes to facing drought condition challenges by evaluating the potential benefits of introducing desalinated water into a subtropical lake. We studied two alternatives and found that desalinated water addition under a nutrient-rich water alternative provided increased benefits. While storing desalinated water in a natural lake is currently a solution unique to Israel (will commence in 2024), water scarcity in the future may invoke such projects in arid and semiarid regions around the world.

### Importance of ensemble modeling and a weather generator

Using a model ensemble increases the reliability of the predictions (Moore et al. 2021). It strengthens our confidence in the predictions when the ensemble members agree on trends, for example, the lake physical properties, the epilimnion oxygen and the response to the hypolimnetic withdrawal & AN scenarios. Similarly, the ensemble suggests caution regarding predictions that differ between models, and thus evokes awareness to model weaknesses and prediction uncertainty. Predictions using a single model are blind to structural uncertainty and thus might yield erroneous conclusions (Willcock et al. 2020).

Uncertainty also arises from climate scenarios and variability in meteorological conditions. Here, we chose the extreme RCP 8.5 climate change scenario, but it is uncertain which RCP scenario will materialize. Uncertainties might be different under a climate scenario of lower magnitude. Not only the climate scenario is uncertain but also the meteorological condition variability within each scenario. By using hundreds of meteorological time series produced by a weather generator, we can estimate how this variance affects the lake ecosystem and use the median projection as the most likely outcome. Uncertainty due to meteorological conditions is in some cases considerable. For example, the biomass of Green and NfixCyano phytoplankton groups may vary in the nutrient-rich water scenario, ranging between 0 and 100% relative to the no-action scenario; this uncertainty is portrayed by the 500 realizations. Many studies use a single meteorological time series as forcing data, neglecting the huge variance in meteorological conditions that can occur under one climate scenario. Using a single time series may be an un-representative event, leading to wrong conclusions. The results of this study highlight the importance of using a model ensemble and a weather generator to reveal uncertainty and underscore the most likely outcome.

### Limitations: Uncertainty and simplifications

While the results suggest a course of action, limitations of the projections must be acknowledged. These limitations include uncertainty as seen in the ensemble results and simplifications made by both models. Uncertainty stemming from model structure emerges from the use of ensemble modeling. Divergence between the two models was found in several variables; for example, the effect of lake level resulted in 13 variables out of 20 showing opposite trends. Analysis showed that the reason for the differences between WET and DYCD models stem from both turnover day and oxygen depletion rate in the hypolimnion. These two attributes have opposing effects on the oxygen concentration in the hypolimnion. Both models resulted in the same trend but different magnitude in both attributes. The differences in oxygen levels most probably drove a majority of the other differences observed between trends in the models. These differences between models render the ecosystem response to lake level uncertain.

Simplifications made by both models cannot be revealed by the ensemble so one must be aware of them. The most outstanding simplification of 1D models is that they do not account for spatial variability. Nutrients that flow into Lake Kinneret from the Jordan River are not mixed uniformly into the lake. There is evidence that the spatial pattern of algal blooms follow the Jordan River inflows (Ostrovsky and Yacobi 2009) and develop in the region adjacent to the river inflow into the lake (G. Tibor unpublished data). Thus, in reality, it is possible that increased nutrient loading will produce different bloom intensity than projected by the 1D models. Additional source of uncertainty stems the projection of the Jordan River nutrient concentration (see Appendix S1). Also, both models do not consider how salinity reduction might affect the microbial community. Preliminary studies showed that reduced salinity is not expected to affect the ecosystem considerably, assuming no ions are added (Sukenik et al. 2021). Overall, the uncertainty, revealed by the model ensemble and by the response to the variable meteorological conditions, is considerable and together with the model’s limitations must be acknowledged when interpreting the results. Minding the uncertainty, it is yet plausible that the trends projected by the models will take place.

### Use of KSI

Using a water quality index as one of the model outputs is a novel approach that enables assessing effects of climate on ecosystem health, as a meaningful summary of the effects on specific variables and processes. Use of such an index is beneficial for management purposes as it facilitates decision making. Here, we used the KSI, a Lake Kinneret ecosystem state index (Gal and Zohary 2017), to simplify multifaceted model results. The KSI suggests that the only scenario that is better than not taking any management action is increasing nutrient flow into the lake combined with hypolimnetic withdrawal. This scenario is anticipated to keep the ecosystem closest to desired past conditions under climate change. The simplicity of KSI is also its drawback: some of its components have opposite responses to specific management actions. For example, the Cyano component of KSI contains NfixCyano and CyanoMC; these have contrasting responses to nutrient-rich water addition scenario and so the difference is blurred. Similarly, this scenario keeps the total N close to historical values while the total P moves away from them, so the combined index culminates in a moderate response. The moderate response together with the model uncertainty leads to minimal KSI difference between scenarios.

## Conclusion

Lake managers should decide on a course of action to mitigate the effect of the expected rising temperatures on the Kinneret ecosystem. Through examining several management actions using ensemble modeling and realization of numerous meteorological conditions, we conclude that the release of additional natural waters into the Jordan River is likely to result in counteracting some climate change effects. Hypolimnetic withdrawal might have additional positive effects but may increase lake warming. Despite the above statements, the results convey a great deal of uncertainty. The proposed action has the likelihood of mitigating some climate change effect, while continuous monitoring of the lake condition and updating models should take place to reduce uncertainty and adjust future actions if necessary (Walker et al. 2013).

The plan to produce and store desalinated water in Lake Kinneret presents an opportunity to preserve the Kinneret ecosystem at a stable state in face of climate change. Introducing desalinated water into a lake or reservoir is presently unique to Israel but may be applicable to semiarid regions where droughts are becoming frequent.

## Supplementary Information

Below is the link to the electronic supplementary material.

Supplementary file1 (PDF 1197 KB)
