# Supplementary material for: Assessing alternative lake management actions for climate change adaptation
Source: Ambio. 2024 Jun 14;54(3):416–27. doi: 10.1007/s13280-024-02039-y (PMC11780036; doi:10.1007/s13280-024-02039-y)
Supplement: Supplementary file 1 — Supplementary file1 (PDF 1197 KB) [file 13280_2024_2039_MOESM1_ESM.pdf]

Ambio

Supplementary Information *This supplementary information has not been peer reviewed.*

Title: Assessing alternative lake management actions for climate change adaptation

Shajar Regev[1,2], Yohay Carmel[2], Gideon Gal[1]

[1] Kinneret Limnological Laboratory, Israel Oceanographic and Limnological Research, Migdal 14950000, Israel. [2] Faculty of Civil and Environmental Engineering, The Technion-Israel Institute of Technology, Haifa 3200003, Israel.

Corresponding author: Shajar Regev, email: shajar.regev@ocean.org.il

Appendix S1 – approximation for nutrients in streams

Daily inflows and nutrients from streams are needed as boundary conditions for the models. The Jordan River flow was obtained from the HYMKE based hydrological model. We have analyzed past observations of Jordan River Nutrient concentrations, investigating correlation with flow and seasonality. Lacking better method and resources to develop one, the Jordan River nutrients concentration was calculated as a linear approximation of (1) mean nutrient concentration per day of the year (from historical data) and/or (2) of the flow volume. Similarly, for the Jordan River water temperature we used a linear approximation with two variable: (1) mean water temperature per day of the year and (2) air temperature at Kfar-Blum station, generated by WeatherCop. See Table S1 for the approximation used. The Jordan river constitute about 75% of the flow into Lake Kinneret. Other streams are smaller and thus have small impact. For those smaller streams we used mean per day of the year from historical data for flow, temperature, and nutrient concentration as boundary conditions for the 50-year runs. The decoupling of the smaller streams from meteorological conditions means that 25% of nutrient inflow is constant regardless of rainy or drought years.

In the DJ & DJHW, an addition of 150·106 m3 desalinated water per year is delivered into the Jordan River through the Hula valley. The addition was from February through to June every year. This addition influenced the nutrient load not only because of the higher volume of water but also because concentration is dependent on flow.

Table S1 – Approximation of Nutrients (and temperature) in the Jordan river. Subscript 365 denotes mean nutrient concentration per day of the year. Units of measure: Flow in M^3/s , Nutrients in mg/l .

Variable	Approximation	R^2 fit to observations	Comment
Temperature	$0.63 \cdot Temp_{365} + 0.22 \cdot AirTemp_{Kfar\text{-}Blum} + 2.78$	0.85	
PPOM	0.00578·Flow + 0.015	0.22	
POM	2.63·Flow + 5.81	0.21	
PDOM	$0.00083 \cdot Flow + 0.57 \cdot PDOM_{365} + 0.0027$	0.34	
PO_4	$0.000713 \cdot Flow + 0.935 \cdot PO_{4\ 365} – 0.0086$	0.34	
NDOM	$0.026 \cdot Flow + 0.65 \cdot NDOM_{365} + 0.5$	0.31	
NO_3	$0.028 \cdot Flow + 0.636 \cdot NO_{3\ 365} + 0.478$	0.32	
SiO_2	See (Regev et al. 2023)		Only in WET
NH_4	$NH_{4\ 365}$	0.12	
NPOM	$0.011 \cdot Flow + 1.14 \cdot NPOM_{365} – 0.368$	0.14	
SAL	SAL_{365}	0.2	Only DYCD
DO	DO_{365}	-	Not enough observations. Only DYCD
TOC	TOC_{365}	0.31	Only DYCD
DOC	DOC_{365}	0.44	Only DYCD
pH	pH_{365}	0.22	Only DYCD
SSOL1	PPOM + PDOM + NPOM + NDOM	-	Type 1 suspended solids in DYCD. No observations.

Appendix S2 - Withdrawals calculation

It was assumed that lake level will be managed in future scenarios by water withdrawals from the lake to avoid overflow or low water level, while maintaining natural level fluctuations. To calculate required withdrawals, a preliminary WET model was run for each realization and each scenario. The WET water balance parameters were set to maintain constant level in this preliminary run. The model output contains “residual stream” to compensate for lake level, which is used to calculate the withdrawals. To allow for natural seasonal level fluctuations the withdrawals were summed for the year, then running mean was calculated for 2 years and then distributed according to distribution of withdrawals observation according to Eq. S1

Eq. S1 $$Daily\ distribution = 0.001072237 \cdot sin((\frac{2\pi}{365.25}) \cdot (yday + 73.7)) - 0.002735052$$

Where yday is day of the year. Small correction factors had to be introduced to compensate for differences between WET and DYCD and differences between constant water level (in the

preliminary run) and the variable level. These corrections were different for each scenario in the form of Eq.S2 $withdrawals = resid_{sc} + a_{m,sc} \cdot day + C_{lvl,sc}$. Where $resid_{sc}$ is the residual stream from the preliminary WET run, sc denote that this is per scenario, $a_{m,sc}$ is constant per model and scenario (see Table S2), day is day of the model run starting from 0, $C_{lvl,sc}$ is constant per lake level and scenario (see Table S3). The median of mean annual lake level deviates between scenarios in less than 40 cm. These lake level differences between scenarios are small enough to defuse the effect of lake level on physical properties of the lake.

Table S2 – Correction factor $a_{m,sc}$ for withdrawals

	**Scenarios**		
model	No-action; AN; DJ; DT	DJHW	DTHW
WET	$5.62 \cdot 10^{-5}$	$5.62 \cdot 10^{-5}$	$5.46 \cdot 10^{-5}$
DYCD	$5.85 \cdot 10^{-5}$	$5.89 \cdot 10^{-5}$	$5.87 \cdot 10^{-5}$

Table S3 - Correction factor $C_{lvl,sc}$ for withdrawals

	**Scenarios**			
Lake level	No-action; AN	DJ; DT	DJHW	DTHW
L1	-0.012	-0.591	-0.457	-0.454
L2	-0.71	-1.56	-1.419	-1.417

Appendix S3 – introducing desalinated water into lake Kinneret

To date (2024), about 57% of Israel's freshwater water supply originates from water desalination plants. Description of the Israel's desalination experience with a focus on the associated energy requirements, environmental policies and perspectives of decision makers can be found in (Tal 2018), and a more general discussion of water-energy considerations in the middle-east can be found in (Weinthal and Sowers 2020).

The decision to bring desalinated water into Lake Kinneret is a result of a number of policy considerations: (1) The eastern and upper Galilee regions rely currently only on natural waters for consumption by the local population and irrigation. These needs are increasing while rainfall in the region declines (Tal 2019). So an alternative water supply is needed. (2) Israel supplies water to the Kingdom of Jordan, currently all of it from Lake Kinneret. There is an increasing demand by the Kingdom to increase the volumes of water supplied to them by Israel.

(3) The Israeli Water Authority sees the lake as a strategic source of water and hence aims to maintain the lake at a high level. (4) Currently Israel has surplus capacity of desalinated water production.

Though desalination has a high energy demand (3.5 kWh per cubic meter, about 0.6$ per cubic meter), for all the above considerations, the decision was made to bring desalinated water into the lake. The project was completed (up to Tsalmon stream) and is expected to commence operation in 2025 though it is dependent on precipitation as average or above average rainfall will delay the initiation of flow of desalinated water into the lake. Several studies investigating the expected effects of introducing desalinated water into the lake were conducted by the Kinneret Limnological Laboratory, IOLR, (Amitay 2021; Sukenik et al. 2021; Amitay et al. 2022; Stein 2023). The project of desalinated water introduction into Lake Kinneret is also called "National Carrier Flow Reversal Project", additional information on the project can be found in (Mekorot 2024).

Appendix S4 – Lake level effect

All management action scenarios were run once while the lake was maintained at high lake level (L1, initial lake level -211 masl) and once when the lake was 3 m lower (L2, initial lake level -214 masl). Maintaining low lake level (L2) reduced residence time by about 0.75 year in the final year of the simulation compared to L1 (Figure S1). Lake level had an effect on a number of variables, but the affects were consistent across scenarios. We therefore provide only the results of lake level effect on the no-action scenario. Summer epilimnion temperature was higher at the low lake level scenarios (L2) compared to high level scenarios (L1), but in winter these results were reversed, and the water was colder with a lower lake level (result not shown). Overall, L2 resulted in a 0.2ºC colder epilimnion water relative to L1 (Figure S1). Both models suggested similar results for the epilimnion but not for the hypolimnion. According to WET model, starting from the turnover of the second year, the hypolimnion was colder in L2 relative to L1 while . According to the DYCD model projected the opposite, the hypolimnion was warmer in L2 relative to L1 by an annual mean 0.2ºC, and in fall-winter – by 0.5ºC. The warmer hypolimnion projected by DYCD is resulted in an earlier turnover (by 11 days), and a longer mixed period. An earlier turnover day was also projected by the WET model, but only by 3 days (Figure S2). The models also diverged in prediction of oxygen trends due to lake level effect. WET suggested that time to oxygen depletion is shorter in L2 because the total oxygen content in the hypolimnion is lower as water volume is lower. Ammonium increased in L2 both in the epilimnion (10%) and hypolimnion (20%). The DYCD model also

predicted quicker oxygen depletion, but to a smaller degree than WET. Since turnover day was earlier, overall hypolimnetic oxygen was slightly higher at a lower lake level. Less phosphorus and less ammonium in winter-spring was predicted, by the DYCD model, at a low lake level (L2). The lower phosphorus in winter caused a decrease in the winter blooming phytoplankton, Diatoms and CyanoMC by 25% and 30% respectively. On the other hand, the WET model projected an increase of CyanoMC by 20% in L2 and minor effects on other phytoplankton groups. Both models agreed that the N:P ratio would increase in L2 by 9 to 15%.

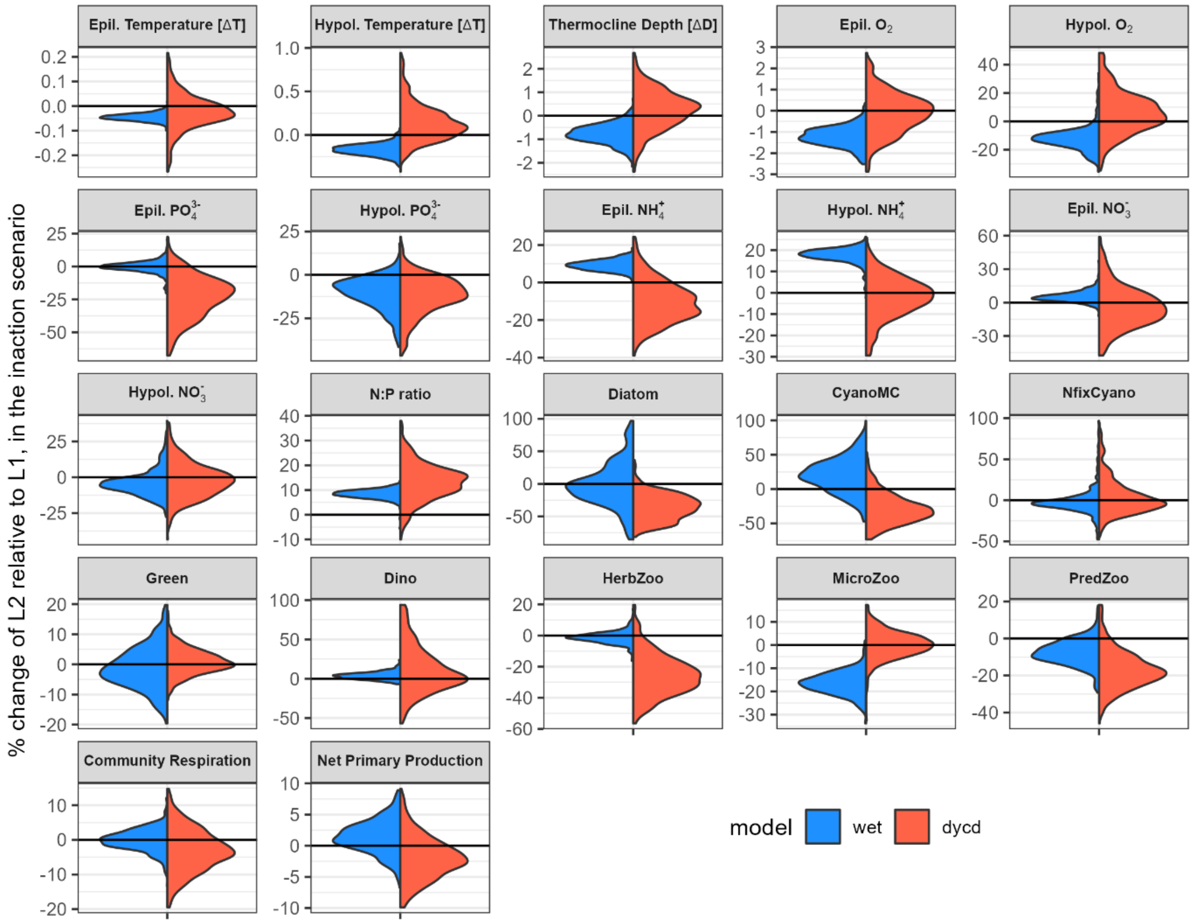

Figure S1 - Lake level affect - change in low lake level relative to high in the no-action scenario. All variables are in % change except for temperature and thermocline that are in difference. Note differences in scale for the various variables. For visibility, density is scaled to have the same width.

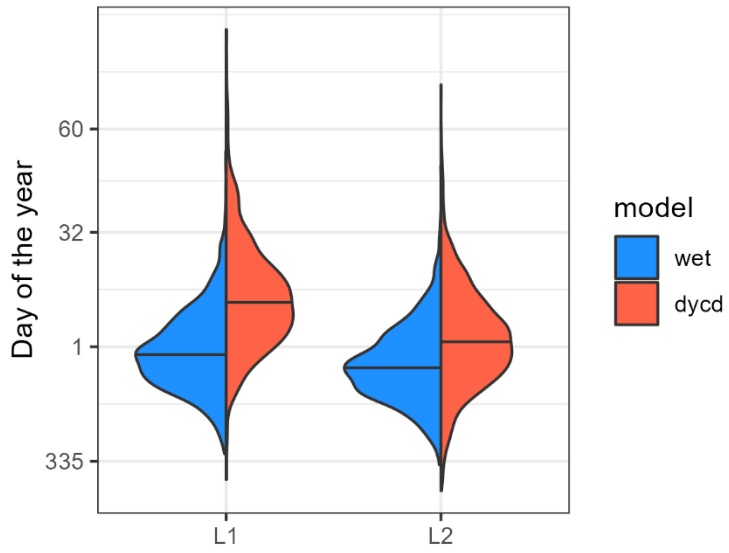

Figure S2 - Lake water column turnover day, the difference between lake levels and models. Line represents the median value.

Analysis showed that the reason for the differences between WET and DYCD models stem from both turnover day and depletion rate of dissolved oxygen in the hypolimnion. These two attributes have opposing effects on the oxygen concentration in the hypolimnion. Both models resulted in the same trend but different magnitude in both attributes. The longer mixed period at low lake level, for example, was much more pronounced in the DYCD model, and caused higher dissolved oxygen levels. Faster hypolimnetic oxygen depletion rate was pronounced in the WET model and resulted in a lower oxygen concentration. These differences resulted in a divergence between the models in hypolimnetic oxygen concentrations. The differences in oxygen levels most probably drove a majority of the other differences observed between trends in the models. These differences between models render the ecosystem response to lake level uncertain.

Appendix S5 – KSI calculations and components affecting the KSI score

The analysis included calculation of the Kinneret ecosystem Sustainability Index, KSI, (Gal and Zohary 2017), which was developed based on comparing 9 ecosystem variables to a reference state. The 9 variables are: (1) Secchi depth (S) (2) Total suspended solids (TSS) (3) TN (4) TP (5) Chlorophyl *a* (Chla) (6) Primary production (PP) (7) Cyanobacteria biomass

(Cyano) (8) *Peridinium gatunense* biomass (Perid) (9) Predatory zooplankton (Zoop). Variables S and TSS are not an output of the DYCD model and was approximated from other output variables using the results from (Gilboa et al. 2009). These results translate into the following equations:

$$S = 2.844 + \frac{0.1386}{Kd} - 0.0062 \cdot Chl$$

$$TSS = 10^{(1.265 - 1.534 \cdot \log(S))}$$

Where Kd is Extinction coefficient, Chl is chlorophyl a.

To build the ranking function for each KSI component, values from an unchanging climate scenario runs were used, the same way it was used in the original method (median values for winter-spring and summer-fall). This is done separately for WET and DYCD models. The reference values used for the KSI calculation were obtained from simulated data of an unchanging climate scenario, based on the 1990 to 2020 period, while the original reference was obtained from 1969–1992 in which conditions were substantially different. This is the reason that the KSI values in this study are considerably higher than the values obtained from the on-going Lake Kinneret monitoring program and those reported by (Gal and Zohary 2017). The non-linearity of the ranking function for the KSI components and the weighted average that is used to calculate the KSI out of its components – makes the order of calculation yield different results. For Figure S3 we have chosen the following calculation order:

1. Calculate variables values for each model, scenario, and realizations per month in 49th year.
2. Calculate percentiles (10%, median, 90%) over the 500 realizations.
3. Calculate ranking of each variable (values between 10-100). The reference values are median values for winter-spring and summer-fall of a baseline scenario in which meteorological conditions are based on the conditions of the period 1990-2020 (baseline scenario in Regev et al. 2024). This is done for each model, scenario, and months.
4. Calculate Ensemble value (mean of WET and DYCD)
5. Calculate mean of the 12 month for all KSI components for each model and scenario.
6. Calculate KSI (weighted average of the 9 variables)

In addition to providing a single gauge, facilitating decision making, the KSI enables quick understanding of which components are contributing to that single index. The components most affecting the index are Total N, Total P, Chlorophyl a & Cyanobacteria (Figure S3).

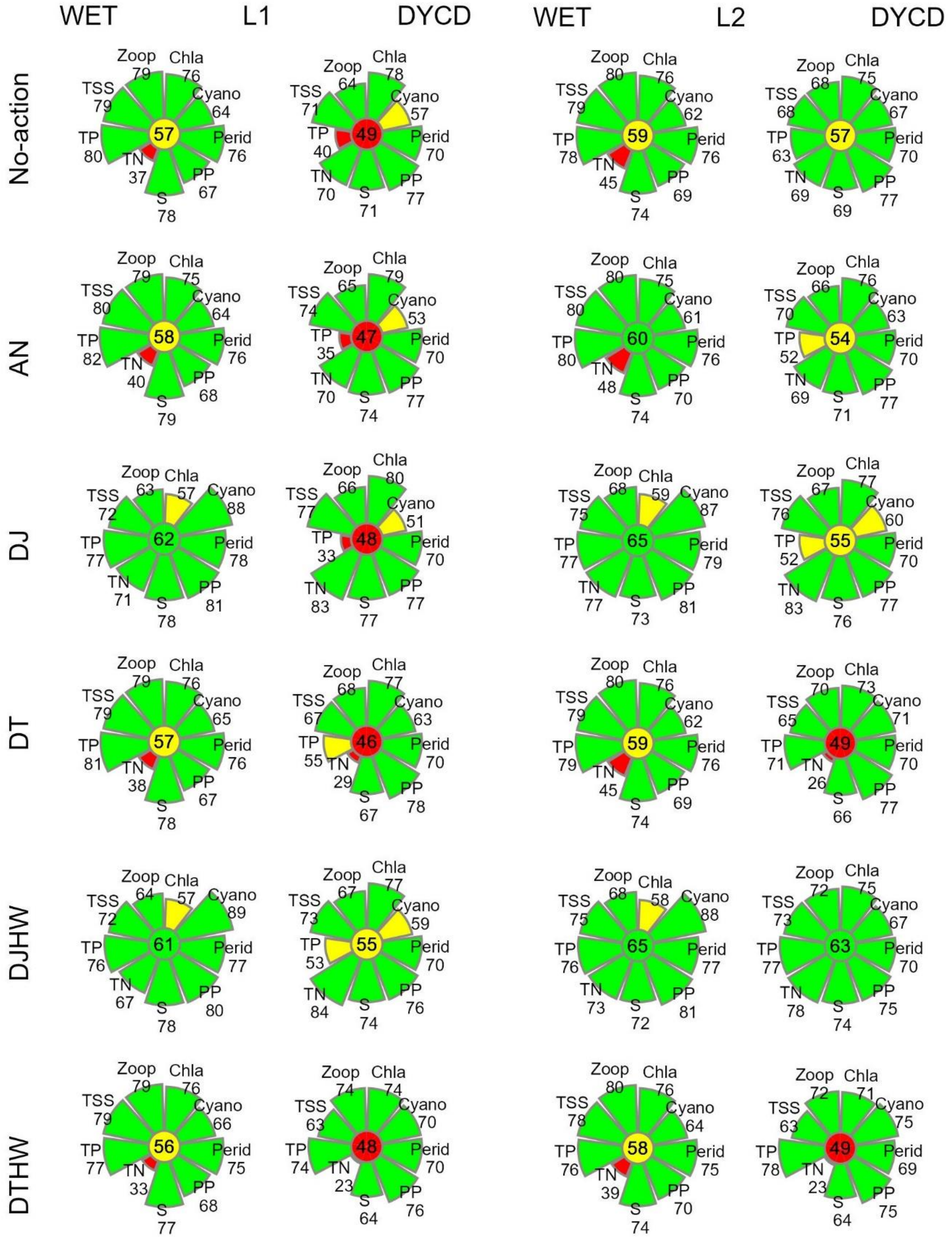

Figure S3 - KSI components at the last year for all scenarios, lake level and models. Green colored components have a rank value >60, yellow 60≥rank≥50 and red for a rank<50.

KSI additional figures

For Figure S4 & Figure S5A the KSI calculation order was different than above since the 500 realizations variability was preserved. Calculation order:

1. Calculate variables values for each model, scenario, and realizations per month in 49th year.
2. Calculate ranking of each variable (values between 10-100). This is done for each realization, model, scenario, and months.
3. Calculate ensemble value (mean of WET and DYCD)
4. Calculate 12 month mean for all KSI components for each realization, model and scenario.
5. Calculate KSI (weighted average of the 9 variables)

The distribution of KSI values over the 500 realizations for the two models, across various scenarios and two lake levels, showed that the variability due to meteorological conditions and between the two models is much higher than the effect of any specific management action (Figure S4). Most KSI values projected by the WET model where above 60 which is considered acceptable (Gal and Zohary 2017), whereas the DYCD model had lower KSI values. DYCD suggested that hypolimnetic withdrawal will have a positive effect because of reduced phosphorus and CyanoMC. Surprisingly, low lake level had a positive effect on KSI; lower TP and a decrease in CyanoMC projected by the DYCD model - improved the KSI. The WET model projected that low lake level would cause an increase in nitrogen; this increase counteracted the climate change effect and positively affected the KSI.

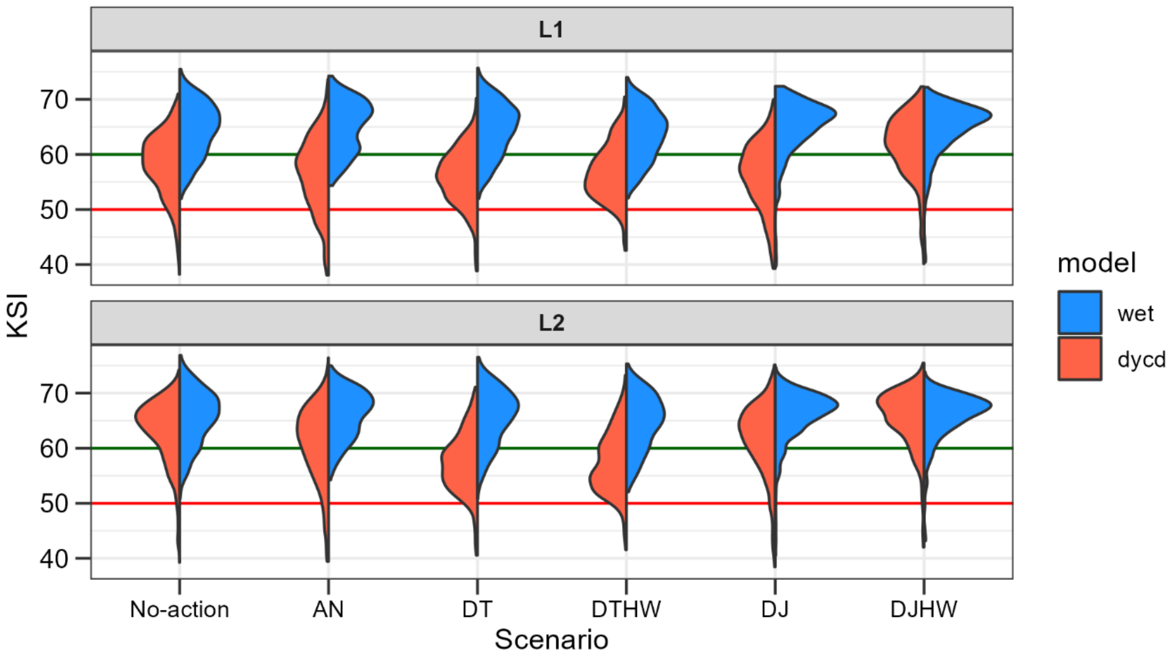

Figure S4 - Distribution of KSI in the last year of model runs, by model and lake level, for the no-action, AN, DJ, DT, DJHW & DTHW scenarios. Top & bottom figure denote Lake levels L1 & L2, respectively. Horizontal lines represent acceptance values – KSI > 60 acceptable state, 60>KSI>50 unacceptable, and KSI<50 highly unacceptable.

Figure S5A shows that releasing natural spring water by bringing desalinated water to the Hula valley, together with hypolimnetic withdrawal (DJHW) is the only scenario that is better than taking no action. "Better" means closer to conditions in the last 30 years. Variability of DT & DTHW is small compared to DJ & DJHW. Overall variability (uncertainty) is high. It is apparent that any mitigation action cannot return the ecosystem condition to a state without climate change. However, when looking at a single variable, for example N fixing cyanobacteria (Figure S5B), the DJ & DJHW management action scenarios can keep this variable close to a no-climate change scenario.

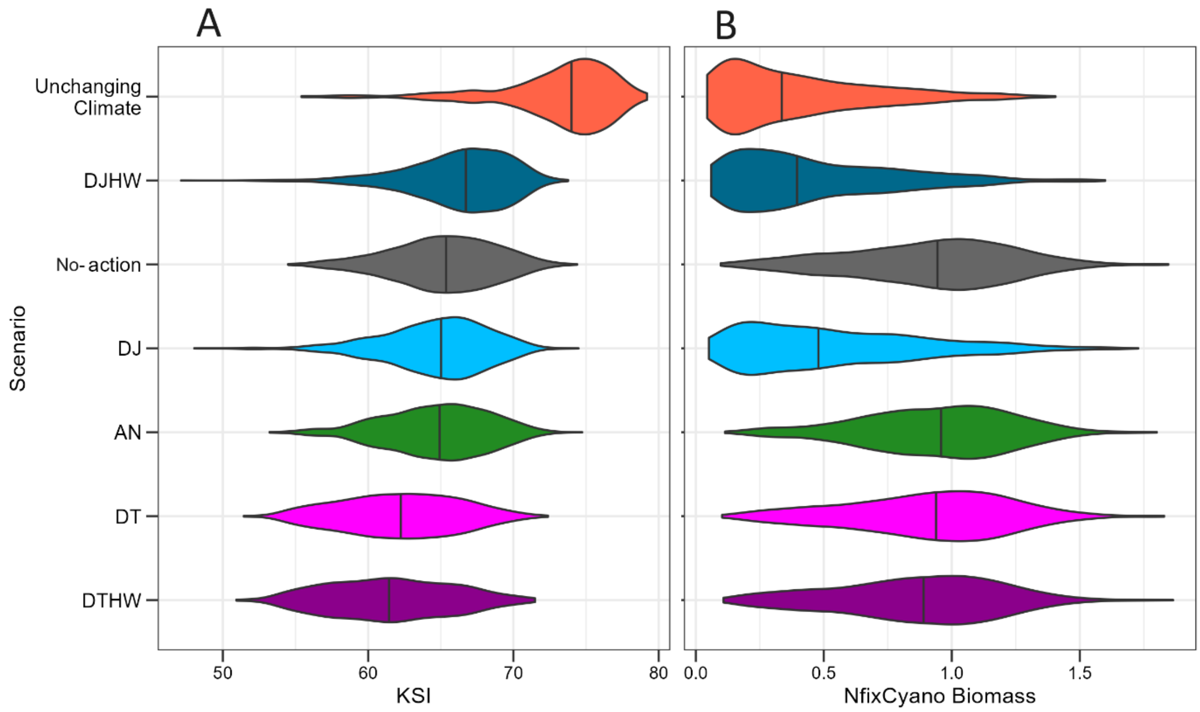

Figure S5 – A (left): KSI ensemble mean. Both lake levels are included. B (right) – N fixing cyanobacteria mean annual biomass in the last year of the model runs. Unchanging Climate scenario is taken from baseline scenario in (Regev et al. 2024). *Black Line represents the median value. All violins have the same area.*

<u>References for all appendices</u>

Amitay Y (2021) Testing the impact of desalinated water discharge into Lake Kinneret by a 3D model. T8-2022 Annual research and monitoring report, chapter 3.8, Kinneret Limnological Laboratory, (In Hebrew)

Amitay Y, Stein S, Koren N, et al (2022) Monitoring and modelling the water addition through Tzalmon stream. T12-2023 Annual research and monitoring report, chapter 3.4, Kinneret Limnological Laboratory, (in Hebrew)

Gal G, Zohary T (2017) Development and application of a sustainability index for a lake ecosystem. Hydrobiologia 800:207–223. doi: 10.1007/s10750-017-3269-1

Gilboa Y, Friedler E, Gal G (2009) Adapting empirical equations to Lake Kinneret data by using three calibration methods. Ecol Modell 220:3291–3300. doi: 10.1016/j.ecolmodel.2009.09.007

Mekorot (2024) National Carrier Flow Reversal Project. https://www.mekorot-int.com/blog/project/national-carrier-flow-reversal-project/. Accessed 16 Apr 2024

Regev S, Carmel Y, Gal G (2023) Using high level validation to increase lake ecosystem model reliability. Environ Model Softw 162:105637. doi: 10.1016/j.envsoft.2023.105637

Regev S, Carmel Y, Schlabing D, Gal G (2024) Climate change impact on sub-tropical lakes – Lake Kinneret as a case study. Sci Total Environ 921:. doi: 10.1016/j.scitotenv.2024.171163

Stein S (2023) Mixing dynamics of stream water into Lake Kinneret through a short-period flow release test. In: EGU23, the 25th EGU General Assembly. Vienna, Austria

Sukenik A, Zohary T, Ninio S, et al (2021) Impact assesment of desalinated water introduction on the lake's biological components. T8-2022 Annual research and monitoring report, chapter 3.9, Kinneret Limnological Laboratory, (In Hebrew)

Tal A (2018) Addressing desalination's carbon footprint: The Israeli experience. Water (Switzerland) 10:. doi: 10.3390/w10020197

Tal A (2019) The implications of climate change driven depletion of Lake Kinneret water levels: the compelling case for climate change-triggered precipitation impact on Lake Kinneret's low water levels. Sci Total Environ 664:1045–1051. doi: 10.1016/j.scitotenv.2019.02.106

Weinthal E, Sowers J (2020) The water-energy nexus in the Middle East: Infrastructure, development, and conflict. Wiley Interdiscip Rev Water 7:. doi: 10.1002/wat2.1437
